# A narrative review of the decade-long integration of professional postgraduate education with standardized residency training in China

**DOI:** 10.3389/fmed.2026.1892608

**Published:** 2026-10-23

**Authors:** Wenda Wang, Xin Zhao, Yi Zhao, Wenwen Chen, Xingcheng Wu

**Affiliations:** Department of Urology, Peking Union Medical College Hospital, Chinese Academy of Medical Sciences & Peking Union Medical College, Beijing, China

**Keywords:** China, dual identity, integration, professional postgraduate education, standardized residency training

## Abstract

Since 2014, China has comprehensively implemented the Standardized Residency Training (SRT) program nationwide, and the integration of professional postgraduate education with SRT has operated for over a decade. This narrative review, based on searches of Wanfang Data and PubMed from January 2015 to December 2025, examines the achievements, persistent challenges, and reform options of this integration system. While the system has shortened medical training from six to three years and enriched clinical exposure, a fundamental structural contradiction persists: the dual identity of professional postgraduates as both “students” and “residents” creates an inherent conflict between clinical practice and academic training. Within the fixed three-year period, postgraduates bear the combined burden of 33 months of clinical rotations, coursework, and thesis research, leaving insufficient protected time for scientific research, compounded by misaligned training objectives, welfare inequity, delayed licensure, and elevated mental health burdens. Enrollment expansion has further strained training capacity and produced crowding-out effects on the broader medical talent pipeline. Implementation-level adjustments can partially alleviate these issues but cannot resolve the core contradiction. Reform should prioritize addressing the dual-identity tension through either flexible decoupling or complete separation of the two pathways, drawing on international experiences.

## 1 Introduction

Currently, in China's clinical medical education, the five-year undergraduate medical program is the most common option offered by the majority of medical schools in China; most undergraduate graduates must complete standardized residency training (SRT) before they can truly practice medicine independently. Since 2014, the Chinese government began to comprehensively promote the SRT system nationwide (1). This system centers on “five-year undergraduate medical education plus three-year SRT or three-year postgraduate education with professional master's degree”, supplemented by “three-year secondary school or junior college medical education plus two-year training for general practitioners” (2). The three-year SRT phase under the “five-year undergraduate medical education plus three-year SRT or three-year postgraduate education with professional master's degree” model includes both pure “residents” and “professional postgraduates”. Additionally, corresponding to “professional postgraduates” are “academic postgraduates”, both with a three-year duration. The distinction lies in the fact that the former undergo SRT concurrently, while the latter complete it after graduating from postgraduate studies.

Since the nationwide implementation of the SRT program, the integration of professional postgraduate education with SRT (hereinafter referred to as the “integration system”) has been in operation for over a decade. During this period, numerous scholars have discussed this system and identified existing issues. To further evaluate the potential challenges of this integration system, we conducted this review.

## 2 Literature search strategy

Given the narrative nature of this review, we did not pursue a systematic review and therefore did not follow the PRISMA guidelines. Nevertheless, to achieve comprehensive coverage of relevant publications, we conducted a literature search across the following electronic databases.

Database searches were conducted on March 12th, 2026. The Chinese database used was Wanfang Data, while the English database was PubMed. The search terms were (standardized residency training) and (professional postgraduate education) with the Boolean operator “AND”. The language or publication-type restrictions were not applied. The date range was from January 1st, 2015 to December 31st, 2025. We assessed the abstracts for potential inclusion in the study, and then the full texts were reviewed. The reference lists were also examined for potential additional studies. The original articles and reviews discussing the issues associated with the integration of professional postgraduate education and SRT were selected. We excluded the following articles: (1) those that did not discuss the issues related to the integration of professional postgraduate education and SRT; (2) those related to traditional Chinese medicine programs; (3) those published before 2015; (4) those were not in Chinese or English; (5) Articles in Chinese which were not included in core journals (those included in Peking University Core Periodical Catalogue, Chinese Science Citation Database, Chinese Social Sciences Citation Index, China Science and Technology Paper Citation Database, or China Medical and Health Core Periodical Catalogue; to achieve a more comprehensive integration of references, journals covered in any historical edition of the aforementioned core journal databases were defined as core journals in this study).

## 3 Policy origins and intended benefits of integration

To standardize the quality of physicians in China, the SRT program, primarily led by the National Health and Family Planning Commission (NHFPC), was officially launched nationwide in 2014 (1). The Chinese Medical Doctor Association (CMDA), directly administered by the NHFPC, was entrusted with overseeing operational details of SRT, including the development of training curricula and accreditation standards (3). This system contributes to improving the overall competence and homogeneity of the clinical physician workforce in China (4). SRT aims to develop clinicians with sound ethics and solid clinical competence, who possess high-level ideological awareness, skilled professional capacity, and good work conduct, and are competent to independently provide standardized diagnosis and treatment for common diseases in their specialty. Six dimensions constitute its core competencies: professionalism, clinical capability, patien-centered care, communication and collaboration, teaching proficiency, and lifelong learning and self-improvement (5). The standardized training duration is set at three years. SRT base accreditation follows the *Standards for the Accreditation of Standardized Resident Training Bases (Trial)* and its revised *Standards for Standardized Resident Training Bases (2022 Edition) (*6, 7). Accreditation of institutional training bases mainly draws on hospitals' basic conditions, training facilities and equipment, and training-system development, while specialty-specific base accreditation considers faculty qualifications and department construction, et al (6, 7).

At present, within China's clinical medical education system, the five-year Bachelor of Medicine program is the most common track offered by the majority of Chinese medical schools, upon completion of which students are awarded a Bachelor of Medicine degree. Holders of this degree are eligible to participate in the three-year SRT. On the other hand, graduates may also pursue a master's degree after obtaining their bachelor's degree. Master's programs are further divided into professional master's and academic master's, both with a three-year duration. Professional master's candidates complete SRT alongside required clinical-oriented research during their postgraduate studies. By contrast, academic master's students focus primarily on medical research. If graduates of academic programs intend to engage in clinical practice, they are required to complete an additional three-year SRT. Following completion of a master's degree, students may proceed to doctoral studies in medicine and earn a Doctor of Medicine (MD) degree upon graduation. Notably, individuals who have not undergone SRT prior to obtaining their MD still need to complete the three-year SRT as an extra requirement for clinical practice (8, 9).

In addition, several Chinese medical universities currently provide an integrated “5 + 3” program. This continuous pathway combines five-year undergraduate medical education with three-year postgraduate training incorporating SRT, and candidates are exempted from the separate master's-level entrance examination. Furthermore, 14 Chinese universities currently offer an eight-year medical education program; graduates are conferred an MD degree but still must finish SRT before practising medicine. In 2019, Nanjing Medical University launched a nine-year medical education program that integrates undergraduate, master's, doctoral education and SRT (9). Moreover, to meet primary-care service demands, an auxiliary pathway exists: three-year secondary-technical or junior-college medical education followed by two-year SRT for general practitioners (2).

Thus commenced the integration of professional postgraduate education with SRT, which has been implemented in China for over a decade. The “integration system” has, to some extent, shortened the duration of medical talent cultivation in China. If postgraduate education and the SRT are delivered separately, the total training period will span six years (three years for postgraduate studies and another three years for residency training), which is equivalent to the length of current academic postgraduate programs. In contrast, the integrated model reduces this period to three years, thereby saving time and costs (10). Meanwhile, the “integration system” can significantly enrich the clinical experience of professional postgraduates (11), improve training efficiency, reduce redundant learning and training, and avoid the waste of educational resources (11, 12). Other reports indicate that the “integration system” can lower employment barriers and enhance graduates' employability. From the perspective of employing hospitals, recruited professional postgraduates have already completed SRT, so they can engage in clinical work immediately upon employment, which reduces hospitals' staffing-related costs (13). However, the “integration system” is unique to China and serves as a transitional model in China's medical education reform (14). The integration process has also revealed numerous persistent issues that remain challenging to resolve.

## 4 Structural overload within a fixed three-year training period

The SRT generally lasts 36 months. For professional postgraduates, their clinical training shall be no less than 33 months. During clinical training, postgraduates shall participate in teaching rounds, mortality and morbidity conference, Mini-lectures and other teaching activities, and take part in clinical supervision for internship students and junior residents (3). During the training period, professional postgraduates shall complete the standardized curriculum required by the SRT. The curricula consist of two categories: general courses (involving laws and regulations, public health, infectious diseases, medical humanities, medical ethics, evidence-based medicine, clinical research and other relevant modules), and specialty-specific courses, which cover knowledge of common and frequent diseases of the specialty, core technical skills, together with assessment, diagnosis and management of critical illnesses (5). Professional postgraduates shall also receive appropriate research training and complete their degree theses. Research training and thesis topics shall be mainly oriented toward clinical problems. The degree thesis may take the form of research reports, summaries of clinical experience, clinical therapeutic effect evaluations, evidence-based studies of specialized literature, literature reviews, or experimental studies addressing clinical-related problems (3).

## 5 Dual identity and fragmented governance

The “integration system” is known as “medicine-education collaboration”. In fact, postgraduate education falls under medical universities and is led by education authorities, with an emphasis on building students' foundational theoretical knowledge of medicine while cultivating their clinical skills. This means completing the master's degree programme constitutes students' primary obligation. SRT represents post-graduate medical education under the oversight of health authorities. It forms a key component of post-graduation education for professional master's students in clinical medicine, whose main objective is to train high-level clinical physicians and improve the quality of medical care (15). Therefore, professional postgraduate students hold dual identities as both “students” and “residents”.

## 6 Consequences for training outcomes, well-being, and equity

The integration of professional postgraduate education and SRT faces deep-seated structural contradictions, with the dual identity of professional postgraduates as both clinical trainees and students serving as the core underlying driver.

### 6.1 Core identity conflict: the fundamental tension between clinical practice and academic training

The dual role of professional postgraduates as both resident physicians and students generates an inherent conflict between clinical practice and scientific research, which constitutes the primary contradiction of the integration system.

#### 6.1.1 Time allocation imbalance: competing demands of clinical rotations and research

The SRT mandates 33 months of clinical rotations, during which postgraduates must fulfill heavy clinical duties while also passing the National Medical Licensing Examination and the SRT completion examination. This schedule leaves insufficient time for dedicated scientific research activities, resulting in low satisfaction with research training effectiveness and academic competency development (10–12, 15–24). Due to clinical rotations across different departments, professional postgraduate students maintain limited contact and insufficient communication with their master tutors. Since a core responsibility of tutors is to guide research training and master's thesis work, this disconnection substantially hinders postgraduates' research progress (20, 22, 25).

Conversely, due to the requirements for scientific research, the quality of clinical skills training for professional postgraduates is also adversely affected (25). This is also reflected in the fact that their scores in clinical practical skills examinations are lower than those in theoretical examinations (26). Some literature has suggested that since professional postgraduates are required to complete their graduation theses, administrators of certain training bases allow them to reduce their clinical rotation time (26).

#### 6.1.2 Misaligned training objectives and evaluation mechanisms

A deeper institutional cause of the clinical-research conflict is the confusion between training objectives for professional and academic postgraduates, paired with a research-centric evaluation system. In terms of training goals, professional postgraduates are meant to acquire clinical experience via SRT and conduct clinical research, while academic postgraduates focus primarily on basic medical research. However, tutor evaluation relies heavily on research outputs, with no consideration given to professional postgraduates' clinical proficiency.

This mismatch leads to excessive requirements for research achievements among professional postgraduates, alongside insufficient attention to clinical training. Given their already limited research time, this pressure may drive postgraduates to meet graduation requirements with superficial research outputs, ultimately resulting in the dual predicament of “weak research capabilities and limited clinical competencies” (14, 26–28).

#### 6.1.3 Curriculum-clinic scheduling conflicts

The dual training mandate also manifests in direct scheduling conflicts between academic courses and clinical rotations. A questionnaire survey of professional postgraduate students found that 41.8% considered the course timetable unreasonable, as courses frequently encroach on ward round time, rest periods, and other scheduled departmental activities (16). Beyond scheduling issues, the curriculum design itself has structural flaws: within limited timeframes, it fails to comprehensively cover professional knowledge, research training, and humanities education (27). Research guidance courses in particular exhibit notable shortcomings, failing to adequately support graduate students in conducting research and contributing to their relatively low research proficiency.

### 6.2 Systemic support deficits: spillover effects of dual identity on welfare and wellbeing

The dual student-trainee identity extends beyond training conflicts to create gaps in welfare protection, professional status, and mental health support, which in turn feed back to undermine training quality and patient safety.

#### 6.2.1 Compensation inequity and ambiguous status

Lower remuneration is a widespread issue stemming from the ambiguous identity of professional postgraduates (20, 23, 25, 28–30). As master's degree candidates, they maintain student status under the jurisdiction of educational authorities, meaning they are required to pay tuition and receive no salary income, relying solely on postgraduate funding subsidies (25). This has prompted calls for coordinated efforts between national and provincial health and education departments to ensure “equal pay for equal work” for professional postgraduates (29).

#### 6.2.2 Licensing delay and patient distrust

Due to their student status, professional postgraduates typically obtain their practicing physician license only during their first year of SRT. This lag in formal qualification often leads to distrust from patients (27), which further limits trainees' opportunities to practice clinical skills and erodes the quality of clinical training.

#### 6.2.3 Excessive workload and mental health burden

The dual demands of clinical rotations and academic training impose significant stress on professional postgraduates (24). Studies show that three-quarters of them work 50–60 h weekly, balancing research and coursework with clinical duties and resulting in insufficient rest (10, 24). Such pressure may create negative feedback loops affecting clinical rotations, potentially compromising medical practice and patient care quality (24), while research demands further exacerbate their stress levels (22).

Compared to social SRT candidates, professional postgraduates exhibit significantly higher depression scores and perceived stress levels, with academic, scientific research, and employment pressures being the primary stressors (31, 32). Excessive work pressure can lead to burnout among resident physicians (32), affecting their physical and mental health and also impacting medical safety (33).

### 6.3 Structural design flaws in training architecture

Beyond identity conflicts, the integration system suffers from inherent design flaws in discipline structure and training duration, which limit the adaptability and sufficiency of SRT.

#### 6.3.1 Discipline specialization: premature narrowing vs. generic rotation

When applying for professional postgraduate programs, majors are usually designated down to the third-level discipline. For instance, Clinical Medicine is a first-level discipline; Surgery and Internal Medicine belong to second-level disciplines; while General Surgery and Urology are categorized as third-level disciplines. Premature focus on a specific specialty can impair training in basic knowledge and skills, and hinder the comprehensive cultivation of competencies required for residents' career development (25).

Paradoxically, however, the system fails to provide adequate training in the specific third-level discipline. The 33-month rotation program usually offers no targeted emphasis regardless of trainees' research focus, resulting in insufficient exposure to their specific discipline (11, 19, 20). Consequently, upon graduation, while employers tend to classify professional postgraduates into tertiary disciplines, their specialized clinical competencies fail to meet the requirements for immediate clinical practice (11, 21).

#### 6.3.2 Rigid program duration and inadequate specialist training

Another critical structural issue is the conflict between SRT requirements and the fixed duration of postgraduate education (28). Since postgraduate programs last three years, it is impossible to adjust the duration of SRT according to different specialties. The SRT is effectively “bound” to the three-year postgraduate timeline, preventing adjustments to its duration based on professional needs as seen in other countries. For example, SRT spans 3–8 years across various specialties in the United States (34).

Although it is recognized that a three-year training period still fails to meet clinical demands, adjusting the duration of SRT is impractical due to constraints imposed by postgraduate education duration. Moreover, standardized specialist training has not yet been widely implemented in China, leaving the inadequacy of SRT under the current integration system unresolved.

### 6.4 Scale-driven systemic crisis: enrollment expansion and capacity shortfalls

Rapid expansion of professional postgraduate enrollment has amplified existing structural contradictions, creating a systemic crisis in training capacity and talent pipeline balance.

#### 6.4.1 Entrance selection mechanism and eroded undergraduate clinical foundation

The current selection mechanism for professional postgraduates does not focus on undergraduates' daily academic performance. As a result, undergraduates tend to neglect their compulsory clinical internships while preparing for the postgraduate entrance examination (PEE) (14, 25, 27).

A study exploring the impact of the PEE on internships conducted interviews with five-year undergraduate students majoring in clinical medicine, who were preparing for the PEE during their clinical internship. All respondents expressed strong subjective willingness to participate in the internship and recognized its importance in familiarizing them with hospital operations, assisting in professional selection, and facilitating a smooth transition to SRT. However, under PEE pressure, they averaged reallocating 60.3% of their internship time to exam preparation, creating a gap between intention and reality (35). Consequently, upon entering the postgraduate stage, they exhibit relatively weak clinical practice skills and must compensate for missing internship components to enhance their clinical competence. This inevitably results in clinical supervisors being hesitant to delegate responsibilities and patients being reluctant to entrust clinical procedures to postgraduates with inadequate clinical skills (14).

#### 6.4.2 Resource constraints and compromised training quality

In recent years, the enrollment scale of professional postgraduate students has gradually expanded. Since the integration system significantly reduces study duration (three years shorter than academic postgraduate programs) and allows graduates to obtain both a postgraduate degree and complete SRT, it has drawn extensive applications from newly graduated undergraduates.

However, the growth rate of professional postgraduates far outpaces that of preceptors and hospital beds. Consequently, SRT for professional postgraduates is inadequately implemented within the integration system. For instance, trainees fail to meet stipulated requirements in terms of the number of managed inpatient beds and clinical skill operations (11, 27).

#### 6.4.3 Crowding-out effects on the broader medical talent pipeline

The rapid expansion of professional postgraduates has two distinct crowding-out effects on the wider medical talent system. First, it affects the quality of candidates for academic postgraduate programs, further hindering the development of innovative scientific research capacity in China's medical field (25). Second, the sharp surge in numbers has limited the capacity of residency training bases to recruit social-source resident physicians. Some training bases even only enroll professional postgraduates for SRT, which severely hinders talent cultivation for medical institutions at all levels (25).

## 7 Why implementation-level adjustments have been insufficient

Faced with the insufficient scientific research training of professional postgraduates under the “integration system”, scholars have put forward corresponding solutions and suggestions. First, it is recommended that each professional postgraduate be assigned two tutors: one clinical tutor and one research tutor, so as to balance the development of both clinical and research competencies of professional postgraduates (36, 37). However, in the admission of professional postgraduates, the supervising tutor is mainly responsible for research training and graduation thesis guidance for professional postgraduates, yet does not directly administer their clinical rotations. Therefore, the dual-tutor system may fail to further enhance research guidance and supervision for postgraduates. Fundamentally, the core bottleneck restricting research performance among professional postgraduates remains the lack of “protected research time”. Incorporating research-time planning into the SRT programme to allocate dedicated research periods for postgraduates has been proposed (20, 24). However, given the fixed total training duration, setting aside exclusive research time will inevitably encroach upon time for clinical training. Hence, rational adjustment of the duration for professional postgraduates merits consideration. Some scholars suggest that professional postgraduates should be offered the option of extending their training period; those unable to complete training within three years may apply for appropriate time extension (20). Second, scholars recommend incorporating research output into the performance appraisal system for SRT bases and tutors. Indicators concerning postgraduates' paper publications shall be included in the regular assessment (36). It is advisable to define explicit minimum requirements for postgraduates' published papers and dissertations, and such requirements should not be set too low (e.g., taking one review article as the threshold) (16, 21). Scholars also propose that tutors should provide standardized guidance throughout clinical research projects, covering the whole workflow including research protocol formulation, ethical application, study implementation, data analysis, and drafting of summary reports. This aims to comprehensively improve postgraduates' clinical research capacity instead of merely satisfying minimum requirements (15). Meanwhile, content related to clinical research methodologies should be optimized in curricula (16).

In terms of curriculum setup, to guarantee the time allocated for clinical rotations, the traditional offline centralized teaching can be transformed into a combination of multiple modes with expanded online course resources. Drawing on foreign experience, unified massive open-online courses (MOOCs) can be developed at the national level (14, 20). In China, graduate school alliances formed by several medical universities have already been established to share open-online courses (14). This approach not only helps balance the time between clinical rotations and coursework, but also standardizes curriculum quality across different SRT bases.

To ensure the quality of SRT clinical training, the SRT system must be strictly implemented. Against the backdrop of expanding numbers of various clinical learners (including SRT trainees, visiting scholars, interns, etc.), it is necessary to guarantee the actual number of in-patient beds managed and clinical skill operation cases completed by professional postgraduates (13). Given the limited bed capacity of SRT bases, resources from collaborative institutions and joint-training programs should be fully utilized. To improve competency-oriented training, some scholars recommend appropriately prolonging rotation time in key departments relevant to the postgraduates' specialty and shortening rotations in other departments. Rational arrangement of rotation departments facilitates professional postgraduates' acquisition of discipline-specific knowledge and skills (11, 19).

With regard to remuneration, the state, universities and SRT bases shall make overall considerations to appropriately improve the salary and benefits of professional postgraduates through multiple channels such as scholarships, grants and work allowances (38). As professional postgraduates are administered by both the Ministry of Education and health authorities, multi-department collaboration is required to optimize their remuneration. It should be clarified that, in addition to postgraduate stipends, SRT bases shall provide performance pay and bonuses, so as to gradually realize “equal pay for equal work” with other resident physicians.

In addition, given the different training objectives for professional and academic postgraduates, it is recommended to optimize the doctoral “application-assessment” selection mechanism and establish two separate selection tracks for professional and academic postgraduates (24).

As mentioned above, concerning the currently-implemented “integration system”, proposals put forward by some scholars can partially improve the existing situation. Measures such as allocating more research time, offering more research-related courses, and establishing research-related assessment requirements may help foster the research competence of professional postgraduates. Optimizing course formats, fully leveraging clinical resources from collaborative institutions, and implementing “equal pay for equal work” can further secure the delivery of clinical rotations for professional postgraduates. To alleviate the clinical training burden of the SRT, management and quality control during medical school internships should be optimized, and systematic, rigorous accreditation of medical schools should be strengthened (39), with the aim of achieving national or regional standardization. This would ensure baseline entry-level competence for professional postgraduates entering SRT and reduce redundant repetitive coursework within SRT. Nevertheless, these adjustments cannot resolve the heavy combined workload of “clinical rotation-research-coursework” imposed by the fixed three-year training cycle. Given unchanged competency standards for professional postgraduates, the effectiveness of such measures may be limited.

## 8 Reform options and their trade-offs

For a long time, the degree-centered evaluation system has led to substantial disparities in clinical competence among physicians, while blurring the boundary between research capacity and clinical practical ability (40). The “integration system” combining SRT with professional postgraduate education is a manifestation of this degree-centered mindset. This integration may further obscure the dividing line between professional practice and academic science (40). Alternative adjustments may be adopted to mitigate the degree-centered paradigm.

First, flexible decoupling may be considered. Residents may complete SRT first and then decide whether to pursue a master's degree. Mutual recognition between SRT curricula and postgraduate credits can be established, and variable training durations may be set for different specialties. This approach offers greater flexibility: it no longer enforces uniform academic standards for all residents. By moderately extending total training duration, it guarantees protected time for both clinical rotations and scientific research. Under this model, clear competency requirements for clinical practice and academic research must still be maintained; otherwise, widely-accepted national quality standards for SRT will be difficult to formulate (40). Supporting national-level standards covering credit mutual recognition, funding guarantees, training transition mechanisms, and quality assurance are required.

Second, complete separation of SRT from postgraduate academic education can clarify the respective training objectives and responsible entities of the two pathways and facilitate specialty-specific training design. However, this model may prolong the overall training timeline, raise personal and public fiscal costs, exacerbate developmental inequities stemming from geographic and socioeconomic backgrounds, and potentially reduce physician supply for primary-care and resource-scarce regions. International SRT systems generally do not integrate with postgraduate degree programmes, and relevant reform experiences can serve as references. Take the United States as an example: its residency training system is uniformly administered by the Accreditation Council for Graduate Medical Education (ACGME), covering SRT entry criteria, training processes, and quality oversight. US residency programmes last 3∼8 years, with the core objective of producing physicians capable of independent patient care upon completion, featuring clear annual tiered training milestones (34). Research requirements for clinical residents in the United States are non-mandatory; residents may engage in research voluntarily according to individual circumstances. Residency training costs in the US are covered by the Centers for Medicare and Medicaid Services (CMS) (41). We summarize the main humanistic and institutional distinction between the Chinese and American SRTs in Table 1 (42–54). Overall, the local optimization of China's SRT system may draw on international experiences in entry-competence assessment, training objectives and content, curriculum design, evaluation and quality control, as well as remuneration safeguards. In this regard, attention should be paid to the core contradiction arising from the “dual identity” of professional postgraduates. In actual clinical work, professional postgraduates are physicians-in-training who also undertake patient management, medical record documentation, doctor-patient communication, ward on-call duties and other tasks, directly contributing to the hospital's healthcare service output; they are not simply students. Classifying professional postgraduates as students lowers the labor cost corresponding to the hospital's clinical work and also gives rise to inequitable treatment. For professional postgraduates, as with other types of resident physicians, compensation commensurate with their labor should be provided, they should have a clear employment status and statutory social insurance coverage, their years of service should be recognized, and they should also enjoy other benefits such as paid leave, occupational protection, and contributions to the housing provident fund. Reform in this area should rely on central or local governments and training hospitals sharing the financial burden jointly.

**Table 1:** The main humanistic and institutional distinction between the Chinese and American SRTs.

Comparison Dimension	United States	China
Legal status and employment contract	Residents are legally defined as employees and physicians-in-training rather than students. Training programs are regulated by state labor laws and the ACGME.	Different kinds of resident physicians: 1. Hospital-sponsored residents (trainees dispatched and funded by their affiliated hospitals, who must return to the sponsoring institution after training): labor relations remain with original employers. A tripartite training agreement is signed among the trainee, training base and sending hospital; no employment contract is concluded with the training base. 2. Self-sponsored residents(individuals recruited directly by the training hospital with no prior employment affiliation, who seek employment independently upon completion): may sign employment contracts with training bases to establish formal labor relationships. 3. Integrated postgraduates: sign a tripartite agreement with the university and base, establishing no labor relationship.
Remuneration and annual pay progression	Resident physician compensation is largely standardized nationwide. AAMC 2025 average stipend: PGY-1 about $68,166; PGY-2 about $70,499; PGY-3 about $73,301; senior PGY-8 about $94,215, increasing about 2%–5% per year. Throughout residency, salaries are set at a level slightly above the local average personal income.	Central government subsidy: RMB 30,000 per resident per year (about two-thirds to the trainee, one-third to the base and faculty), with provincial matching and hospital/sending-hospital top-ups. Moreover, professional postgraduates who undergo SRT concurrently are not eligible for the dedicated stipends provided to regular residents, and their actual income is generally lower than that of self-sponsored and hospital-sponsored residents at the same training site.
Statutory social insurance, health insurance and pension coverage	As hospital employees, residents are mandatorily covered under FICA (Social Security and Medicare) with employer matching contributions. Hospitals provide group health-insurance plans. Retirement benefits combine Social Security and hospital-sponsored retirement schemes.	Self-sponsored residents: recent rules require the training base to pay the “five social insurances and housing fund”; hospital-sponsored residents: the sending hospital continues to pay wages, social insurance, and housing fund; integrated postgraduates, as university students, are covered by student health insurance and do not contribute to urban employee social insurance.
Paid annual leave, parental leave and sick leave	ACGME requires at least one full day off in seven; vacation is typically 3–4 weeks per year; a minimum of six paid weeks off for medical, parental, and caregiver leave.	Beyond national statutory holidays, provinces generally cap annual combined leave at 15 days; excess time extends training (including maternity leave and sick leave).
Occupational injury and professional-liability protection	Work-related injuries are covered under state workers' compensation statutes. Training hospitals maintain centralized malpractice insurance covering residents for clinical activities during training.	Self-sponsored residents, as parties to labor contracts, have work-injury insurance by law; hospital-sponsored residents are covered by the sending hospital; medical liability insurance is purchased uniformly by the hospital, but there is no national rule allocating liability for trainees' clinical acts; post-exposure protocols exist, but the work-injury recognition route does not apply to postgraduates.
Protection of educational time and working-hour regulation	ACGME imposes binding working-hour limits: continuous duty ≤ 24 h; weekly working-hour cap of 80 h with at least one full rest day per week. Protected educational time is mandatory. Clinical service may not displace educational activities; non-compliant programs risk loss of accreditation.	National residency policies offer working-hour recommendations, without unified mandatory hour caps or independent third-party oversight. Heavy clinical workload and night shifts are common in front-line rotations. Protected educational time is often compromised by clinical duties, and implementation varies greatly across training sites.
Funding responsibilities of government, insurers and training hospitals	Funding for GME is provided through programs of the United States Department of Health and Human Services (HHS), the United States Department of Veterans Affairs (VA), and the United States Department of Defense, particularly the Medicare Program, the Medicaid Program, and private funds from individual hospitals.	RMB 30,000 per resident per year provided by central government subsidy, supplemented by provincial and some municipal funding; training hospitals cover the balance, position subsidies, performance pay, housing, and faculty costs; the sending hospital covers base wages and social insurance for hospital-sponsored trainees; integrated postgraduates are funded through graduate-education channels and do not receive the central residency subsidy; insurers do not directly fund training.

AAMC, The Association of American Medical Colleges; ACGME, Accreditation Council for Graduate Medical Education; FICA, the Federal Insurance Contributions Act; GME, graduate medical education; PGY-1, post graduate year-1.

## 9 Research and policy-evaluation agenda

Before selecting an appropriate reform pathway, several key contextual factors must also be taken into account, including the differences in status and competence among professional postgraduates, academic postgraduates, and socially recruited residents, the costs of training, and the variation in economic development across regions. Existing single-center work is suggestive but inconclusive: clinical postgraduates report a lower quality of working life than self-sponsored residents (32), while academic postgraduates show stronger research ability (55); however, such comparisons should be grounded in standardized measures of clinical competence and research literacy. Second, objective workload and compensation data (payroll, time-motion, and electronic-record measures) should replace self-report, given mean working times near 80 h per week and low, uneven pay (32, 56). Third, mental-health outcomes-burnout, depression, and turnover intention-must be tracked prospectively and by trainee category, since burnout already affects roughly three-fifths of residents, depressive symptoms about one in four, and nearly half report high turnover intention, potentially even more so under the combined clinical-research-coursework load (57, 58). Fourth, the training costs of each pathway must be micro-costed from government, hospital, and trainee perspectives to judge the fiscal feasibility and distributive effects of decoupling or separation. Fifth, specialty-specific requirements should inform variable-duration design rather than imposing a uniform three-year model (34). Sixth, workforce retention and distribution should be followed through administrative data linkage, given the low retention in rural primary care after compulsory service (57, 59). Seventh, regional and socioeconomic impacts must be evaluated, because pay, hours, and educational debt already vary by region and may deter students from disadvantaged backgrounds (56, 59). Only by fully taking China's national context into account can the choice among these training pathways be guided by evidence on clinical competence, trainee well-being, training costs, and workforce equity, rather than relying solely on international experience.

## 10 Conclusion

Over the past decade, integrating professional postgraduate education with SRT has shortened training and enriched clinical exposure, yet deep structural tensions persist. The core contradiction lies in the dual identity of professional postgraduates—as both students and residents—which forces them to balance 33 months of clinical rotations, coursework, and thesis research within a fixed three-year window, producing an unsustainable workload that undermines both research competency and clinical skill depth. This conflict extends into welfare inequity, delayed licensure, elevated burnout, and, amid enrollment expansion, strained training capacity and crowding-out effects on the talent pipeline. While proposed implementation-level adjustments offer partial relief, they cannot resolve the fundamental contradiction as long as the training period and competency standards remain fixed. Reform should confront the dual-identity issue directly-either through flexible decoupling with credit mutual recognition or complete separation of SRT from postgraduate education-while ensuring trainees receive fair compensation, clear employment status, and occupational protection, drawing lessons from international systems.

## Data Availability

The original contributions presented in the study are included in the article/Supplementary Material, further inquiries can be directed to the corresponding author.
